# From the host's point of view: Effects of variation in burying beetle brood care and brood size on the interaction with parasitic mites

**DOI:** 10.1371/journal.pone.0228047

**Published:** 2020-01-21

**Authors:** Petra Schedwill, Sophia Paschkewitz, Heide Teubner, Nadine Steinmetz, Volker Nehring

**Affiliations:** Evolutionary Biology & Ecology, Institute of Biology I, University of Freiburg, Freiburg, Germany; Universidade de São Paulo, BRAZIL

## Abstract

The fitness and virulence of parasites is often determined by how many resources they can wrangle out of their hosts. Host defenses that help to keep resources from the parasites will then reduce virulence and parasite fitness. Here, we study whether host brood care and brood size regulation can protect host fitness and harm a parasite. We use the biparental brood-caring burying beetle *Nicrophorus vespilloides* and its phoretic *Poecilochirus carabi* mites as a model. Since paternal brood care does not seem to benefit the offspring in a clean laboratory setting, the male presence has been suggested to strengthen the defense against parasites. We manipulated male presence and found no effect on the fitness of the parasitic mites or the beetle offspring. We further manipulated beetle brood size and found larger broods to reduce parasite fitness. The specific pattern we observed suggests that beetle larvae are strong competitors and consume the carrion resource before all parasites develop. They thus starve the parasites. These results shed new light on the observation that the parasites appear to reduce host brood size early on–potentially to avert later competition their offspring might have to face.

## Introduction

Host-parasite interactions are often tugs-of-war for resources. Each symbiont tries to secure as much as possible of the resources for its own reproduction. To this end they often limit the other symbiont's resource use. For example, parasites may castrate hosts to prevent them from using energy for egg production. Instead, the parasites manipulate hosts to invest energy into somatic growth–and the body serves as an energy store until the parasites later use it for their own reproduction. The hosts, in turn, may begin reproduction earlier when infected, to divert as many resources as possible into own offspring before castration is successful [1].

When parasites threaten to take away resources from host offspring, parental care can be a successful means to limit the impact of parasites [2]. Parental care is diverse in form, ranging from selection and preparation of oviposition sites, through guarding eggs, embryos, and hatchlings, to feeding offspring [3–5]. Parental care is costly to the parents and thus limited. There are rare cases of biparental brood care. This should evolve when two parents are better in caring than one, and when mating opportunities are rare [6]. In some systems, male assistance in brood care is essential for offspring survival [7–11], but surprisingly often the direct gains of biparental over uniparental brood care appear marginal [12], which makes one wonder how biparental care could evolve in the first place.

We studied the interaction between parental care and the impact of parasites in *Nicrophorus vespilloides* burying beetles. The beetles reproduce on small vertebrate carcasses [13], which represent a valuable but ephemeral resource. Competition for this resource is intense. Adult beetles often get only one chance to reproduce on a carcass in their entire lifetime [14]. Beetles fight for the resource and the bigger beetle usually wins [15–17]. *Nicrophorus vespilloides* is known for its biparental brood care [15]. The pair provides brood care through three crucial behaviors: The beetles 1) hide and defend the carcass from competitors and parasites (including microorganisms) [14,15], they 2) optimize the trade-off between offspring number and size by regulating egg and larval numbers [13,14,18–20], and 3) they directly feed the larvae [15,21].

Eggert et al. [22] showed that beetle larvae that do not receive parental care grow smaller and are more likely to die. Single parents, on the other hand, are able to raise their young at least as successful as a pair [23–27]. Males in parental pairs appear to hold back on care [12], but may help by guarding offspring and resource [13]. Both parental beetles will consume some part of the resource [25,26,28]. This leads not only to a conflict between the parental beetles, and between parents and offspring, but also increases competition among offspring [12,24,29–31]. Each offspring individual tries to increase its body weight and size by consuming more than its fair share of the resource, to become competitive when it comes to monopolizing resources as an adult [15–17]. Furthermore, there are opportunity costs to parental care because parents cannot reproduce again while caring for brood [14], and investment into parental care constrains future reproductive output [32].

Besides other silphids, there are smaller organisms such as nematodes, flies, and mites that compete for the carcass. Here, we analyze the effect of mites of the genus *Poecilochirus*, which live phoretically on *N*. *vespilloides* and related species, and reproduce along with the beetles on the carcass. Under certain conditions, the beetles might profit from the association with mites: Mites kill nematodes or flies, which are strong competitors of burying beetles [33,34]. Mites can also raise the body temperature of beetles, which makes them more competitive, an effect that is limited to beetles of small body size [35]. In most studies, however, the effect of mites on beetles is found to be negative: The mites reduce the male beetle's life span [36]. This cost borne by the male may indicate that he invests into the defense against mites. Mites also seem to weaken the effect of brood regulation by the parents [30]. The regulation of brood size is crucial because it allows the parents to optimize the trade-off between individual offspring size (and hence their chances in fights for resources) and the number of offspring. Mites can predate on beetle eggs and larvae [37–40] and compete with beetle parents and offspring for the resource [22,29,41,42]. The latter statement is mostly based on the observation that the numbers of mite and beetle offspring (or beetle brood weight as an alternative measure of beetle fitness) are negatively correlated [30,41,42]. Experimental evidence for any directionality in this effect is lacking. Interestingly, increasing the available resources does not reduce the competition but strengthens the negative association between mite and beetle fitness [42]. In invertebrates, parasites often benefit disproportionally much from increased host nutrition because they often manage to consume host resources quicker than the hosts themselves [43,44]. Again, it is not always clear whether the parasites “steal” the energy from their hosts, or whether they merely profit from superfluous energy the hosts cannot consume anyway.

So far, studies on the phoretic mites of burying beetles typically focused on the effects that mites might have on the beetles [34,37,39–41]. In this study we took a different point of view and analyzed in how far variation in beetle brood care would affect mite fitness, with a focus on male brood care and brood size regulation. We tested two hypotheses: 1) Biparental brood care protects beetles against the mites, so that in biparental broods, beetle fitness is higher and mite fitness is lower than in uniparental broods. 2) If the negative fitness interaction between mites and beetles is due to competition for resources, experimentally reducing beetle brood size should increase mite fitness because the beetles will use fewer resources. This effect should be stronger when beetle broods are reduced in size early, before the beetle larvae begin to feed on the carcass and thus remove resources.

## Material and methods

### Beetle and mite maintenance

*Nicrophorus vespilloides* beetles and their phoretic *Poecilochirus carabi* mites were captured in the Mooswald forest (48°2' N, 7°50' E; permission for access by Forstamt Freiburg) north of Freiburg im Breisgau, Germany, and laboratory lines were established under standardized conditions. We bred beetles in containers filled with moist peat on a dead mouse. No siblings or half-siblings were allowed to mate. Beetles were kept in plastic containers filled with moist peat in groups of up to five same-sex siblings and fed twice a week with one beheaded mealworm per beetle. The containers were kept in climate chambers of 20°C in a dark-light-cycle of 8/16h.

Mites were bred without beetles in groups of 15–20 individuals in a container with moist peat and a piece of fresh cow liver. Afterwards, mites were kept on beetles (for at least three days) as described above. No permits were required for the described study, which complied with all relevant regulations.

### Uniparental vs. biparental brood care

Beetles and mites were allowed to breed in peat-filled plant pots (diameter 18 cm, height 16 cm) that trapped beetles leaving the brood. We set up beetles in two different treatments, either as pairs (n = 62) or as single mated females (n = 64), and added either ten (per treatment n = 22), 30 (per treatment n = 20), or no mites (biparental n = 20, uniparental n = 22) to the replicates. Field-caught beetles typically carry around 10–20 mites [33,45,46].

One day before starting the experiment, beetle pairs were set up for copulation in smaller container (10 x 10 x 5 cm). The following day the male was removed for setting up uniparental broods. A dead mouse (reared in the University of Freiburg animal facilities, sacrificed by freezing after anesthesia by CO_2_) of 10 g was given to the beetles and together they were transferred to the plant pot, mites were added, and the pot was covered. Pots were stored in a climate chamber at 20°C with an 8/16h dark-light-cycle. One day later, when the carcass was buried, the setup was closed with another inverted pot. After three days, the exit of the device was opened, so that beetles that left the carcass would be trapped (see [17]). Traps were surveyed twice a day. Mites sitting on trapped beetles were counted. Twenty-one days after starting the experiment, the peat was carefully searched for beetle pupae or remaining mites in the peat. Beetle pupae and mites were counted, and pupae were weighed.

All data were analyzed in R (Version 3.5.1). We analyzed the effects of two predictors, number of parents (uniparental, biparental) and initial mite dose (zero to thirty mites), and their interaction, on beetle fitness measured as total brood weight and offspring number with general linear models with a quasipoisson distribution. We analyzed the effects on mite fitness (two different measures: total offspring number, and number of mite offspring that left the carcass with the parental beetles) in the same way, but here we excluded all replicates to which we had initially not added any mites. We obtained p-values for contrasts using the summary function. Effect sizes and test statistics are listed in S1 Table. To test for homogeneity of variance we conducted Fligner-Kileen tests.

### Beetle brood size regulation

**Fig 1:**
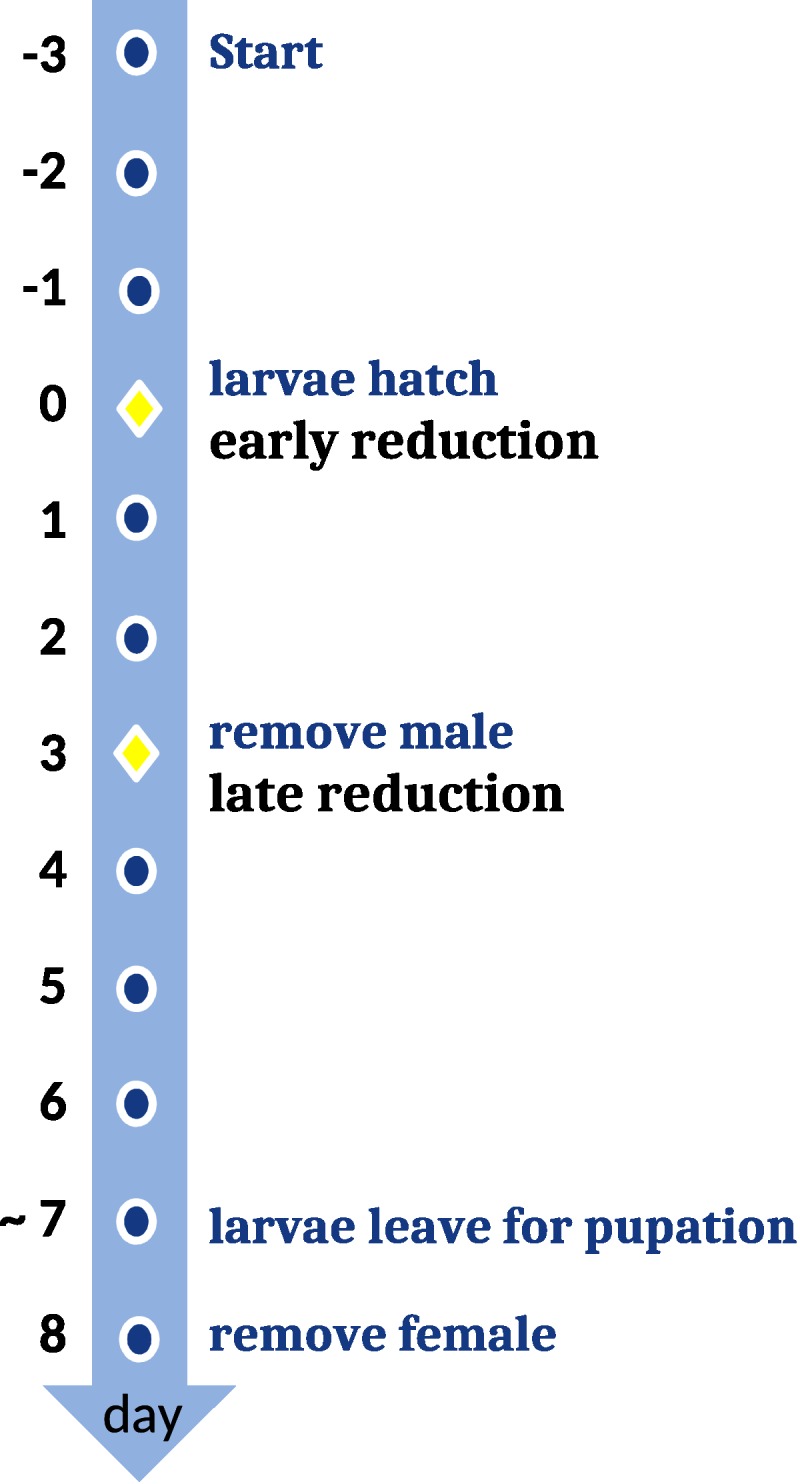
Chronological procedure for the beetle brood size experiment. In the early reduction treatment, we started larval regulation directly after the first larvae appeared on the carcass. Brood size in the late reduction treatment was reduced three days later.

We let pairs of beetles reproduce in small containers (10 x 10 x 5 cm) instead of plant pots, because the small containers allowed us to better access broods to experimentally the beetle brood size to seven larvae. The reduction occurred either when the larvae first arrived at the carcass (Fig 1; early reduction, n = 28) or three days later when the larvae already had consumed a considerable proportion of the carcass (late reduction, n = 31). These replicates were compared with broods in which we manipulated the larvae in the same way but did not reduce brood size (control, n = 33).

One day before the experiment started, beetle pairs were combined in containers with a moist paper tissue and some dead mealworms. On the next day, 15 mites were added to each beetle pair and mites and beetles were transferred to a breeding container filled with moist peat and a dead mouse (frozen “Springer” mice, purchased from frostfutter.de, mean weight 14.4 g, minimum 11.8 g, maximum 16.6 g; the carcass weight did not differ between treatments, linear model p > 0.4, see S1 Table). After the beetles buried the mouse, container were stored in a dark climate chamber at 20°C. Mite dose and carcass size were larger than in the first experiment because some of our experiments indicated that the competition between mites and beetles is highest with these adjusted parameters [42].

We surveyed the broods twice a day, in the morning and in the evening, and reduced beetle brood size were indicated. Surveys were conducted in the dark under a red light source. The carcass including beetle larvae was transferred to a petri dish, while the two parent beetles stayed in the container. All larvae were removed from the carcass with soft forceps and counted. After repositioning the larvae back onto the carcass, we waited for one minute for the larvae to orientate themselves on the carcass and begin to feed, before placing the carcass back into the container.

In the early reduction treatment, the number of larvae was reduced to seven as soon as the larvae arrived at the carcass. In the late reduction treatment, we counted the larvae and placed them back on the carcass during the first three days (initial brood size of late reduction replicates), unless there were more than 21 larvae: then we removed as many larvae as needed to achieve a brood size of 21 individuals. Three days after the first larvae had appeared, we reduced brood size by only placing seven of the larvae back onto the carcass. In the control treatment, the brood size was measured as in the other treatments, but only reduced when there were more than 21 larvae. Here, we never reduced the brood size any further and recorded brood size when we transferred the larvae to new containers for pupation. In two of the replicates (one in the control, one in the late reduction treatment), one larva was likely overlooked during surveys, leading to a (initial) brood size of 22 larvae.

On the third day after the first larvae appeared, when we also reduced brood size in the late reduction treatment, the male beetle was removed from the breeding container. Males typically leave the brood around this time in unmanipulated broods [41]. In small containers, they might disturb the brood when left in the container for longer. Mite offspring were removed from the male beetle with CO_2_ and forceps, and counted. When the larvae abandoned the carcass for pupation, we waited for another 12 hours before removing the female beetle and transferring the larvae to new containers for pupation (10 x 10 x 6 cm, up to 12 larvae per container), stored in a dark climate chamber at 20°C. Mites on the female were removed and counted. After removing remains of the carcass from the breeding container, the female was returned to the container to collect mites that might have been left behind in the peat. Once a day, the female was taken out of the container and new mites on it were removed and counted. After two days of no new mites appearing on the female, the replicate was stopped. Approximately one week after the larvae left the carcass, pupation had taken place. We carefully removed peat and mites, which we counted. We summed up all mites that developed, and only the mites that left the resource on the parental beetles, as two alternative measures of mite fitness. Beetle pupae were counted and weighed.

Statistical analyses were conducted with general linear models as mentioned above, with treatment and carcass weight as predictors (S1 Table). In an additional model, we analyzed the effect of the initial brood size, treatment, and their interaction on the number of mites that developed. The scatterplot was drawn with the “car” package [47].

## Results

### Uniparental vs. biparental brood care

When beetle or mite reproduction failed, we excluded the replicates from any further analysis. Failed beetle reproduction was defined as beetles having no offspring (biparental: 3 replicates, uniparental: 9 replicates) or fewer than 4 larvae (uniparental: 1 replicate); failed mite reproduction meant no mites were present on beetles or their offspring (uniparental: 1 replicate). This resulted in the analysis of 88.9% of 126 initial replicates of the brood care manipulation experiment.

**Fig 2:**
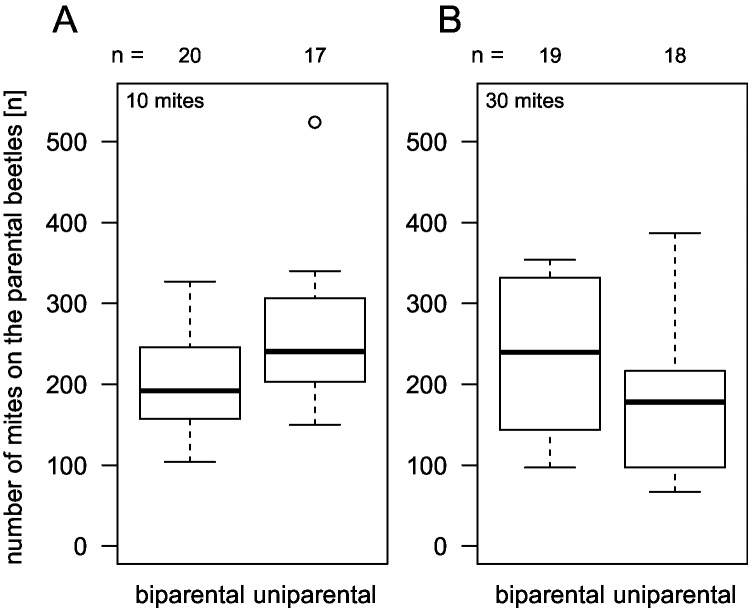
**The number of mites that left the carcasses on the parental beetles was not affected by the number of caring parents.** (p = 0.39), neither at an initial mite dose of 10 (A), nor 30 (B) mites (interaction mite dose x parent number p = 0.44). More mites developed when the initial mite dose was larger (p < 0.001). Boxplots depict median (thick line), interquartile range (box), minimum and maximum. Numbers are sample sizes.

In replicates with successful reproduction, the number of mites that developed on a carcass was larger when the initial mite dose was larger (n = 47, Wald test p < 0.001; details in S1 Table). There was neither an effect of biparental brood care by itself (p = 0.21) nor an interaction of parent number and initial mite dose (p = 0.53) on the total number of mite offspring that developed. The pattern was similar when we only took mites into account that left the carcass with the parental beetles (Fig 2; no effect of biparental care p = 0.39, increase with mite dose p < 0.001).

**Fig 3:**
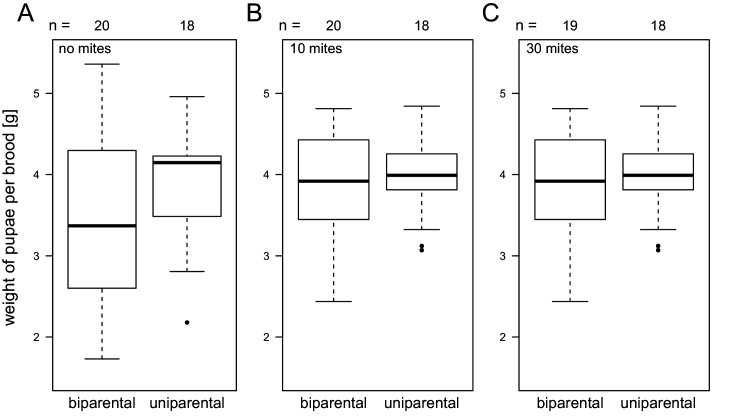
Total beetle brood weight was not affected by the presence of the beetle males (p = 0.09) at any mite dose (interaction p = 0.27). The initial mite dose was 0 (A), 10 (B), or 30 (C) mites, and did not affect beetle brood weight either (p = 0.83). Boxplots depict median (thick line), interquartile range (box), minimum and maximum. Numbers are sample sizes.

There was no strong effect of biparental brood care (n = 112, Wald test p = 0.09), mite dose (p = 0.82), or their interaction (p = 0.27) on the total beetle brood weight or the number of beetle offspring (all p > 0.2, details in S1 Table; Fig 3). However, the variation in brood weight was lower in broods cared for by females only, in particular in the absence of mites, due to no replicates yielding extremely low brood weights (Fligner-Kileen test for homogeneity of variances; no mites χ^2^ = 4.3, p = 0.04; 10 mites χ^2^ = 2.5, p = 0.12; 30 mites χ^2^ = 0.3, p = 0.58).

### Beetle brood size regulation

In the brood size experiment we were restrictive in data allowance for analysis, to ensure that the brood size indeed differed between treatments: Replicates of the early reduction treatment had to have either 6 or 7 larvae pupating, while replicates in the control and late reduction treatment needed at least 12 larvae (initial number of larvae, before reduction in the late treatment took place). This resulted in an analysis of 72.0% of the 92 replicates that were initially set up.

In broods with only 6 or 7 larvae, the mean pupal weight was higher than in control broods of 12 to 22 larvae (control: median = 226 mg, n = 21, early reduction: median = 269 mg, n = 23; gaussian glm, p < 0.001). Pupal weight in broods with late reduction, with a final number of 7 pupae, did not differ from the control treatment (late reduction: median = 240 mg, n = 15; Wald test, t = 1.48; p = 0.15).

**Fig 4:**
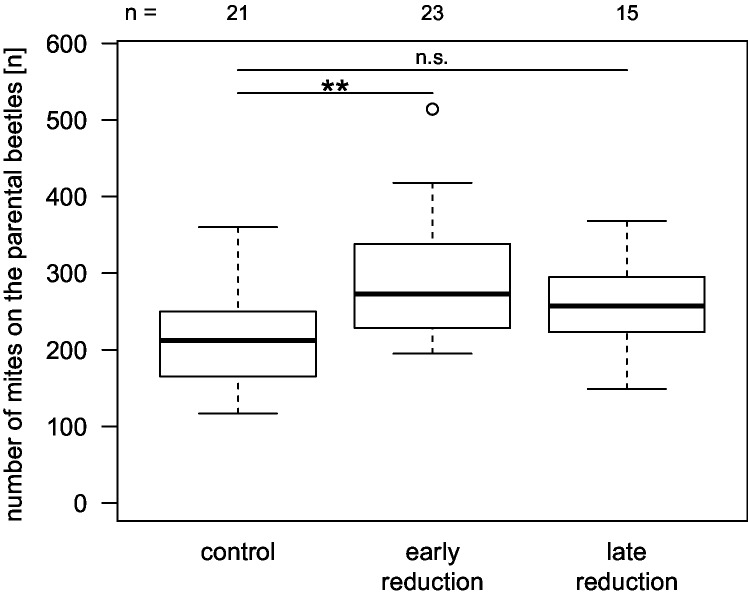
The number of mites that left the carcass with the parental beetles depended on the experimental manipulation of beetle brood size. We experimentally controlled the size of the beetle broods to an average brood size of 12–22 (control) or 7 larvae (reduction). In the low brood size treatments, we removed excessive larvae either when they first arrived at the carcass (early reduction) or three days later (late reduction). Boxplots depict median (thick line), interquartile range (box), minimum and maximum. Numbers are sample sizes; Wald test, ** p < 0.01, n.s. p > 0.05).

When we experimentally reduced the beetle brood size directly after the larvae had hatched (early reduction), more mites developed than in the control broods (n = 21–23 per treatment; Wald test, t = 2.00; p = 0.05). This effect was not evident when we reduced the larval number after the larvae had already fed on the carcass for three days (late reduction; n = 15–21 per treatment; Wald test, t = 0.99; p = 0.33). The average number of mites that developed in the late reduction treatment was in between the numbers from the control and the early reduction treatment (Fig 4). We obtained similar results when analyzing the number of only those mites that left the carcass with the parental beetles (Fig 4; early reduction p < 0.01, late reduction p = 0.11; for details see S1 Table).

**Fig 5:**
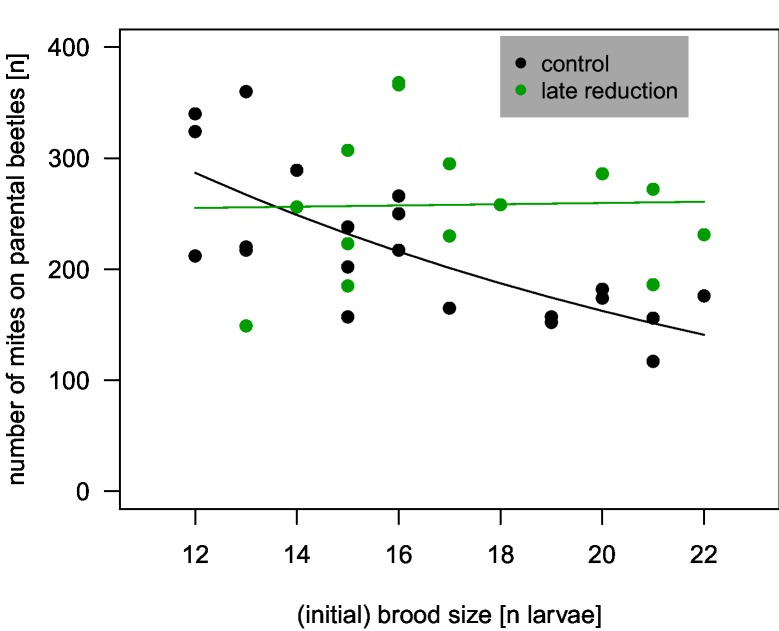
Initial beetle brood size and mites that left the carcass with the parental beetles. The number of mites that developed on carcasses depended on the number of beetle larvae in control replicates of normal size (control treatment, p < 0.001). The effect was weaker when brood size was reduced three days after the larvae hatched (late reduction treatment, n = 15, interaction treatment x brood size p < 0.05). Points depict individual replicates, lines are regression lines.

The larger the brood size in the control treatment, the fewer mites developed and left the carcass on the parental beetles (Fig 5; S1 Table; total mite number p < 0.05, mites on beetles p < 0.001). This effect was weaker or absent in the late reduction treatment (interaction brood size x treatment, total mite number p = 0.15, mites on beetles p < 0.05).

## Discussion

Previous studies have mostly focused on the effects of phoretic mites on their burying beetle hosts, finding negative effects of mites due to mite predation on beetle eggs and larvae and competition with beetle larvae [37,39–42]. In our experiments, we experimentally tested whether variation in beetle brood care would affect mite fitness (offspring number) and found that it does not matter for the mites whether beetle broods are cared for by one or two beetle parents. Biparental care did also not increase beetle fitness (offspring number or total brood mass), so that we conclude that male brood care does not defend the beetles against their mite parasites. We did find that an experimental reduction in beetle brood size increased mite fitness, supporting the idea that mite and beetle offspring compete for resources. The negative relationship between mite and beetle fitness could thus either be caused by random variation in the number of beetle offspring or by excessive predation of mites on beetle eggs. Part of the effect appears to manifest itself late in the development, since a reduction three days after the beetle larvae hatched still could rescue the negative effects of large beetle broods on mite fitness.

### Uniparental vs. biparental brood care

In our experiments, single parents were able to raise their young just as successful as pairs, which mirrors the results of most previous studies that were conducted without testing for the effect of parasitic mites [12,23,24]. We did not find strong effects of biparental brood care on the mites' brood success either. Due to findings that males of the related species *Nicrophorus orbicollis* improve reproductive success under interspecific competition [48] we had hypothesized that *N*. *vespilloides* males might protect the brood from parasites, too. Taken together, our results suggest that this is not the case since beetles did not benefit and mites did not suffer from biparental brood care.

### Beetle brood size regulation

The mites depend on the beetles, as the latter function as a means of transport for the mites and make the carcasses accessible for the mites. While these established effects create a net positive outcome of the interaction with beetles for the mites, several studies demonstrated negative fitness correlations between beetles and mites once on the resource [37,39–41]. Previous explanations for these effects focused on the mites, which appear to compete for carrion and to predate on beetle eggs and larvae [37,39–41]. Here, we took a different point of view and could experimentally show that beetle brood size negatively affects mite fitness: The more beetle larvae develop on a carcass, the fewer mites can develop. Hence, part of the variation in brood success of the mites can be caused by variation in beetle brood size. Interestingly, we could rescue mite fitness by a relatively late reduction of beetle brood size, after the larvae had already grown and thus consumed a considerable part of the resource. This late reduction neutralized the negative effect of beetle brood size on mite fitness.

The late-acting effects of beetle brood size on mite fitness may be informative about the mechanism of the interference. Female mites lay eggs once, over a period of a few days [49,50], and die afterwards, so that continued oviposition after our experimental brood size reduction is unlikely. Since the mites are semelparous, they have no reason to hold back any resources and likely lay the maximum number of eggs possible, always resulting in equally high densities of mite larvae on the carcass in all treatments. Consequently, some mite offspring in the control treatments might die before developing into deuteronymphs. This could happen because 1) beetle parents or larvae consume mites, which to our knowledge has never been observed, 2) beetles harm or kill developing mites otherwise, e.g. through mechanical interference when mites are fragile just after moulting, or 3) mites may starve. There is strong resource competition among beetles and among mites [14,19,42], so that the third scenario is very likely. By experimentally reducing beetle brood size, we reduce competition, leading to higher mite fitness in these treatments. This would mean that the negative correlation between host and parasite fitness, often interpreted as virulence, may in some cases be the consequence of parasite-independent variation in resource consumption by hosts. Such an effect may be one reason why increasing host resources cause higher virulence in some, but lower virulence in other taxa [43].

Now that we have established how beetle brood size can control mite fitness, the question remains why beetle brood size itself is so variable that strong correlations between mite and beetle fitness can be observed even in unmanipulated experiments [39,41], giving the impression that sometimes the beetles, and other times the mites, get the upper hand in the battle for resources. While many factors may affect clutch size and hatching success of the burying beetles (e.g. quality of the parental beetles, microbiome), part of this variation may also be due to the mites themselves: mite adults consume beetle eggs [37] and have been observed to kill young beetle larvae [40]. There are probably direct nutritional benefits from predation for the mites, but given that early in the development the carcass is a relatively large resource, these benefits can hardly be significant. At the same time, mites can reduce beetle brood size by predation, so that the mite parents that predate on beetle eggs and larvae preemptively reduce the competition for carrion that their offspring will face later on.

## Conclusion

Through competition, beetles and mites pose selection pressures on each other. While parental care might sometimes evolve to keep parasites at bay, we found no evidence for male beetles defending the beetle brood against parasitic mites. We did find that smaller beetle broods are benefiting mite fitness, an effect that arose in a late stage of beetle and mite development. The most likely explanation is that large beetle broods consume so much of the resource that later developing mites run out of resource before their development is complete. The mite parents may counteract this by predating on beetle eggs and larvae, and thereby reduce the competition their offspring will face before it even occurs. Our results illustrate how reciprocal fitness effects of parasites and hosts on each other can be to a large extend governed by the competition for common resources.

## Supporting information

S1 Table Detailed test statistics for all general linear models. (PDF)

S2 Table Raw data of the uniparental vs. biparental brood care experiment. (CSV)

S3 Table Raw data of the brood size manipulation experiment. Treatments are control (C), early reduction (ER), or late reduction (LR).
(CSV)
