# Supplementary material for: From the host's point of view: Effects of variation in burying beetle brood care and brood size on the interaction with parasitic mites
Source: PLoS One. 2020 Jan 21;15(1):e0228047. doi: 10.1371/journal.pone.0228047 (PMC6974135; doi:10.1371/journal.pone.0228047)
Supplement: S1 Table — (PDF) [file pone.0228047.s001.pdf]

Experiment: bi- vs uniparental brood care

response variable	predictor	contrast	Estimate	Std Error	t value	p-value
total number of mite offspring	number of parents	intercept: biparental	4.71	0.17		
		uniparental	0.30	0.24	1.26	0.21
	mite dose		0.04	0.01	5.26	< 0.001
	dose x parent no		-0.006	0.009	-0.63	0.53
number of mite offspring on parental beetles	number of parents	intercept: biparental	4.47	0.29		
		uniparental	0.33	0.38	0.88	0.39
	mite dose		0.04	0.01	3.50	< 0.001
	dose x parent no		-0.01	0.01	-0.77	0.44
total number of beetle offspring	number of parents	intercept: biparental	3.08	0.50		
		uniparental	0.01	0.07	0.17	0.87
	mite dose		-0.004	0.003	-1.22	0.22
	dose x parent no		0.001	0.004	0.22	0.83
total beetle brood weight	number of parents	intercept: biparental	1.29	0.38		
		uniparental	0.09	0.05	1.70	0.09
	mite dose		0.001	0.002	0.22	0.83
	dose x parent no		-0.003	0.003	-1.11	0.27

Experiment: brood size manipulation

response variable	predictor		Estimate	Std Error	t-value	p-value
weight of carcass [g]	manipulation	control	14.39	0.22		
		early reduction	0.15	0.30	0.49	0.62
		late reduction	0.27	0.34	0.79	0.43
mean pupal weight [mg]	weight of carcass [g]		-1.34	3.51	-0.38	0.70
	manipulation	control	246.91	50.84		
		early reduction	34.13	7.95	4.29	< 0.001
		late reduction	13.18	8.93	1.48	0.15
total number of mite offspring	weight of carcass [g]		-0.03	0.04	-0.76	0.45
	manipulation	control	6.31	0.61		
		early reduction	0.19	0.10	2.00	0.05
		late reduction	0.11	0.11	0.99	0.33
number of mite offspring on parental beetles	weight of carcass [g]		0.03	0.04	0.72	0.47
	manipulation	control	5.00	0.54		
		early reduction	0.29	0.09	3.34	< 0.01
		late reduction	0.16	0.10	1.63	0.11

response variable	predictor	Estimate	Std Error	t-value	p-value
number of mite offspring in controls and late reduction treatment	Intercept	6.83	0.76	8.93	< 0.001
	weight of carcass [g]	-0.01	0.05	-0.25	0.81
	number of beetle larvae (before reduction)	-0.05	0.02	-2.22	< 0.05
	treatment late reduction	-0.68	0.55	-1.24	0.23
	number of larvae x treatment	0.05	0.33	1.49	0.15
number of mite offspring on parental beetles in controls and late reduction treatment	Intercept	6.20	0.60	10.33	< 0.001
	weight of carcass [g]	0.03	0.04	0.59	0.56
	number of beetle larvae (before reduction)	-0.07	0.02	-4.15	<0.001
	treatment late reduction	-1.03	0.46	-2.22	<0.05
	number of larvae x treatment	0.07	0.03	2.73	<0.05
